# Crowding alters F-actin secondary structure and hydration

**DOI:** 10.1038/s42003-023-05274-3

**Published:** 2023-09-02

**Authors:** Xiaobing Chen, Steven J. Roeters, Francis Cavanna, José Alvarado, Carlos R. Baiz

**Affiliations:** 1 Department of Chemistry, University of Texas at Austin, Austin, TX USA; 2 Department of Chemistry, Aarhus University, Aarhus, Denmark; 3 Department of Anatomy and Neurosciences, Vrije Universiteit, Amsterdam UMC, Amsterdam, Netherlands; 4 Department of Physics, University of Texas at Austin, Center for Nonlinear Dynamics, Austin, TX USA

**Keywords:** Molecular conformation, Biopolymers in vivo

## Abstract

Actin, an important component of eukaryotic cell cytoskeleton, regulates cell shape and transport. The morphology and biochemical properties of actin filaments are determined by their structure and protein-protein contacts. Crowded environments can organize filaments into bundles, but less is known about how they affect F-actin structure. This study used 2D IR spectroscopy and spectral calculations to examine how crowding and bundling impact the secondary structure and local environments in filaments and weakly or strongly bundled networks. The results reveal that bundling induces changes in actin’s secondary structure, leading to a decrease in *β*-sheet and an increase in loop conformations. Strongly bundled networks exhibit a decrease in backbone solvent exposure, with less perturbed *α*-helices and nearly “locked” *β*-sheets. Similarly, the loops become less hydrated but maintain a dynamic environment. These findings highlight the role of loop structure in actin network morphology and stability under morphology control by PEG.

## Abstract (web summary)

2D IR and spectral calculations reveal how crowding and bundling impact the secondary structure and local environments in actin filaments.

## Introduction

**Fig. 1:**
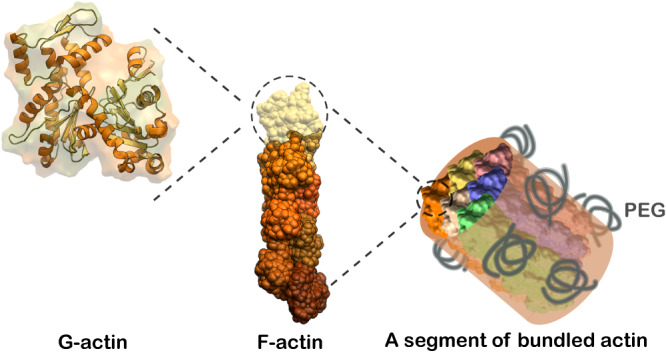
F-actin can form low- to high-order structures. From left to right: G-actin (monomer), F-actin (polymer), and a segment of bundled actin under depletion-driven interactions of polymer PEG. The G-actin and F-actin structures are obtained from PDB files *2HF4* and *6BNO*, respectively.

The actin cytoskeleton is a biopolymer network that provides a scaffold for the structural rigidity of cells^1–4^. Virtually all mammalian cells contain six isoforms of actin, which share 95–99% homology, making actin one of the most evolutionarily conserved proteins in the animal kingdom^5–7^. Actin is essential for a wide range of mechanical functions, including locomotion^8,9^, shape change^10,11^, cell division^4,12^, force exertion^13,14^, mechanosensing^15,16^, and tension homeostasis^17,18^. Actin protein monomers can polymerize into double-helical filaments^19,20^, and these filaments are further organized into a variety of suprafilamentous structures (Fig. 1), such as bundles^19,21,22^, crosslinked filament networks^23,24^, bundle networks^24,25^, and aggregates^26,27^.

The organization of actin filaments into higher-order structures depends on two classes of interaction. First, enthalpic interactions between actin filaments and crosslinks provide a chemical means of controlling the filament organization^22,24,28^. Biological crosslinks are protein molecules containing two or more actin-binding domains^29,30^. There are 13 distinct actin-binding proteins, known as crosslinks^23,31^. Examples include fascin^32,33^, *α*-actinin^33,34^, scruin^35,36^, and ARP2/3 complex^37,38^. Furthermore, actin binds a variety of other actin-binding proteins that do not function as passive crosslinks, such as gelsolin^39,40^, profilin^41,42^, cofilin^43,44^, and myosin^2,3^. Actin-binding proteins bind actin via specific interactions using a variety of actin-binding domains, such as DNase I loop, nucleotide binding cleft, calponin-homology domain, and *β*-trefoil domain^45–49^. Second, a number of physical interactions affect the filament organization. Counterion condensation^25,50^ and depletion interactions^27,51,52^ can also induce bundling. Similarly, steric confinement induces bundling^53^ and affects the filament organization^54,55^. Shear forces can induce nematic patterns^56,57^ and introduce hysteresis^58,59^. Van der Waals attraction between filaments promotes adhesive interfilament interactions that lead to gradual relaxation of prestress^60,61^. Polyethylene glycol (PEG) is known to induce actin bundle formation owing to crowding and depletion interactions between filamentous actin and the polymer^62^. Morphological changes in the F-actin depend on the molecular weight and concentration of the polymer in the solution^63,64^. For instance, 0.1% w/v (or 1 mg/mL) PEG with a molecular weight of 20 kDa can result in strong bundling, whereas PEG with 6 kDa at the same concentration produces weakly bundled networks^65^. Although the bundling degree of actin filaments under morphology control has been established and studied^65^, the effects of bundling degree on actin molecular structure and biochemical properties are still under-explored.

Actin morphology and biochemical properties are closely related. Tension-induced structural changes in filaments affect their binding to certain effector proteins^66,67^. The actin structure is sensitive to the solvent environment, including water and cations, which affects the (de)polymerization and binding capability of actin filaments^68–70^. Therefore, understanding physical properties, such as the structure and dynamics around the backbone, is critical for understanding the biochemical properties of the actin network. Furthermore, studying the impact of morphological control, such as a higher or lower bundling degree, on the physical and biochemical properties of the actin network can inform the development of soft materials using biopolymer assemblies. To this end, ultrafast infrared spectroscopy is utilized to probe the structural and dynamic changes in the actin secondary structure or actin backbone in crowded environments, that is, polymer-crowded aqueous solutions. Ultrafast infrared spectroscopy has a higher spectral resolution and better sensitivity than traditional IR techniques such as FT IR. Additionally, it can perform real-time measurements of non-crystallized actin samples to study transient motions along the protein backbone, which cannot be performed using microscopic techniques.

**Fig. 2:**
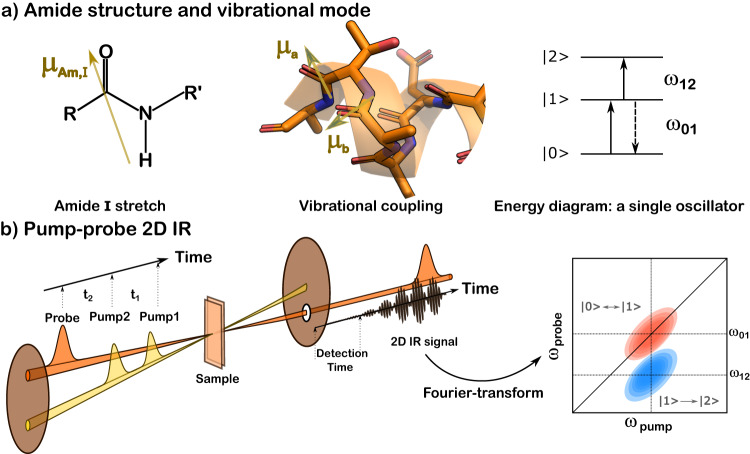
Amide I stretch is an excellent IR probe for the actin structure in 2D IR experiments. **a** Amide unit structure and vibrational mode characteristics. From left to right: transition dipole orientation of amide I stretch; peptide fragment showing coupling among different amide I modes; and energy diagram of a single oscillator. **b** Pump-probe 2D IR. Illustration of pump-probe geometry is adapted from Fig. 1 of the cited publication^104^ (10.1364/OE.471984, open access articles ⓒ 2023 Optica Publishing Group). The color scheme in this illustration has been adjusted to harmonize with the current figure. The cartoon depiction of the 2D IR spectrum is an original creation by the first author. Within this spectrum, the horizontal and vertical axes represent the pump and probe frequencies, respectively, of the amide I mode. The peaks are labeled by the corresponding transition processes, as indicated in the energy diagram above.

This work aims to explore the secondary-structural changes in actin networks and inform their relation to actin functions under morphological control. The relation between morphology, physical properties, and secondary structure is beginning to be explored. To this end, we characterize the secondary structure and local solvent environment across three different morphologies driven by polymer depletion. Actin filaments, weakly bundled networks, and strongly bundled networks are studied using two-dimensional infrared (2D IR) spectroscopy (Fig. 2b) combined with theoretical modeling of residue participation in the vibrational states associated with specific frequency regions. The amide I mode along the backbone of actin (Fig. 2a) is used as an infrared (IR) probe for the actin structure and local environments^71,72^. The 2D IR spectra are interpreted using a vibrational model that relates amide I features to secondary structures, thus providing a molecular description of filament interactions within the different morphologies. In addition, the sub-picosecond dynamics of the solvent molecules are captured by the evolution of spectral lineshape as a function of pump and probe time delays. Results show that spectral features and timescales are similar in filaments and weakly bundled networks, but are very different for strongly bundled networks. Three secondary structures are assigned in the amide I frequency region: *α*-helix, *β*-sheet, and loops/turns. A small yet substantial conformational change from *β*-sheet to loop structure occurs upon the formation of strongly bundled networks. Further analysis of the solvent dynamics reveals the different effects of morphological changes on the local environments of the three different backbones. In summary, this work investigates the structure and dynamics of the actin backbone in different morphologies driven by polymer depletion, offering clues for the correlations between the structure and physical/biochemical properties of supramolecular biopolymer assemblies.

## Results and discussion

### Amide I infrared spectra show multiple secondary structures

**Fig. 3:**
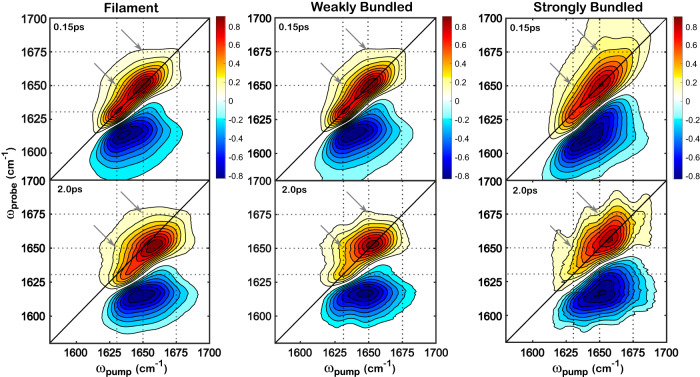
The experimental 2D IR spectra in the amide I stretch region reveal multiple bands corresponding to different secondary structures. Spectra measured at pump-probe delay times (*t*_2_) of 0.15 ps (top) and 2.0 ps (bottom) are shown as examples. Three columns from left to right correspond to the filament, weakly bundled network, and strongly bundled network, respectively, as indicated in the titles. The *X*- and *Y*-axes represent the pump and probe frequencies, respectively. The spectra are normalized to the maxima of the peak intensity for comparison among the different samples. The solid black lines represent the diagonals of the spectra. The dashed lines represent the guidance of the frequency positions (1630, 1650, and 1675 cm^−1^). Arrows indicate the rough positions of the cross peaks. The contour bars to the right of the axes display the current color map and indicate the mapping of data values into the color map. The background of the spectra (peak amplitude below 0.2) is removed for clarity.

The amide I 2D IR spectra of actin samples at two selected pump-probe delay times (*t*_2_) in the filament (no PEG), weakly bundled network (PEG 6 kDa, 0.1% w/v), and strongly bundled network (PEG 20 kDa, 0.1% w/v) are shown in Fig. 3. The amide I stretch lineshapes indicate the backbone structure of the actin networks. The changes in the lineshapes as a function of the delay time (pump-probe delay) report on the local fluctuations and energy redistribution rates among the delocalized amide I modes along the backbone. The positive peaks (shown in red) correspond to the ground state bleach and involve a vibrational transition from the ground to the first excited state, while the negative peaks (shown in blue) correspond to the transition from the first to the second excited state. Two selected delay times (*t*_2_) are included for clarity. Spectra measured at early delay times, such as 0.15 ps, show diagonally elongated peaks due to the high correlation between the pump (*ω*_pump_) and probe (*ω*_probe_) frequencies. The shape of the peaks becomes rounder at later delays due to the gradual decorrelation between the pump and probe frequencies, which results from the frequency fluctuations of individual amide groups, referred to as spectral diffusion, or energy redistribution within the backbone modes. Background noise features become increasingly prominent at longer delay times because of the overall vibrational relaxation of the amide I modes, which further degrades the already weak 2D IR signals at the low actin concentrations used for all samples.

It is evident that more than one band appears along the diagonal in the amide I stretch region, as shown in Fig. 3. All samples exhibit a strong band at 1650 cm^−1^ and a weaker band at 1630 cm^−1^. In addition, the 2D IR lineshape of strongly bundled networks is different from that of filaments or weakly bundled networks. The broad amide I lineshape is known to arise from multiple bands positioned at different frequencies because of the different backbone conformations, where *α*-helix appears around 1650 cm^−1^, *β*-sheet around 1630 cm^−1^, and loops around 1680 cm^−1^^71,73,74^. In Fig. 3, the peak shape in weakly bundled networks does not change significantly compared to the filament, suggesting similar secondary structures between the filament and the network with a low degree of bundling. In contrast, in the spectra of the strongly bundled network, the low-frequency band appears less prominent, while a broader shoulder arises on the blue frequency side compared with the filament and weakly bundled network samples. The lineshape difference of the strongly bundled network sample corresponds to backbone conformational changes and solvent exposure changes under the depletion of PEG, where actin filaments form a high degree of bundles.

In addition to the peaks observed along the diagonal, off-diagonal features are present in the 2DIR spectra that increase in intensity with a longer delay time. The intersections of dashed lines along the off-diagonal direction in Fig. 3 show the approximate positions of the cross peaks. The peak shape can be qualitatively described by lineshapes that span the frequency region around (*ω*_pump_, *ω*_probe_) = (1630, 1650) cm^−1^ in all samples. Off-diagonal peaks are weak at short pump-probe delays but become more pronounced at later delays. These off-diagonal features, or cross-peaks, result from the energy redistribution among the backbone vibrational modes^71,75,76^. For example, if a cross peak is observed at the cross of two modes (*ω*_pump_ and *ω*_probe_), energy flows from one mode at *ω*_pump_ to the other mode at *ω*_probe_. At an early delay time, coupling between the amide I modes of the strong and weaker bands at 1650 and 1630 cm^−1^ shows weak cross-peaks in all samples, whereas at longer delay times, the energy transfer further increases the cross-peak amplitudes. In addition, off-diagonal features around (*ω*_pump_, *ω*_probe_) = (1650, 1675) cm^−1^ are increasingly prominent in strongly bundled networks. Following the same logic, these cross-peaks can be attributed to the coupling of amide I modes between the main band at 1650 cm^−1^ and the increased shoulder band in the 1675 cm^−1^ region of the strongly bundled networks. Notice that the cross peaks in the 2D IR spectrum at 2.0 ps (Fig. 3, bottom right) are obscured by background noise, so they are hard to observe in the positive peaks. However, they appear more evidently in the negative peaks (Supplementary Fig. 1), as seen by the rising shoulders to the side of the diagonal peaks. All findings in the lineshape of the 2D IR spectra are likely to indicate three secondary structures along the backbone of actin biopolymers that undergo structural changes under depletion-driven morphological control.

**Fig. 4:**
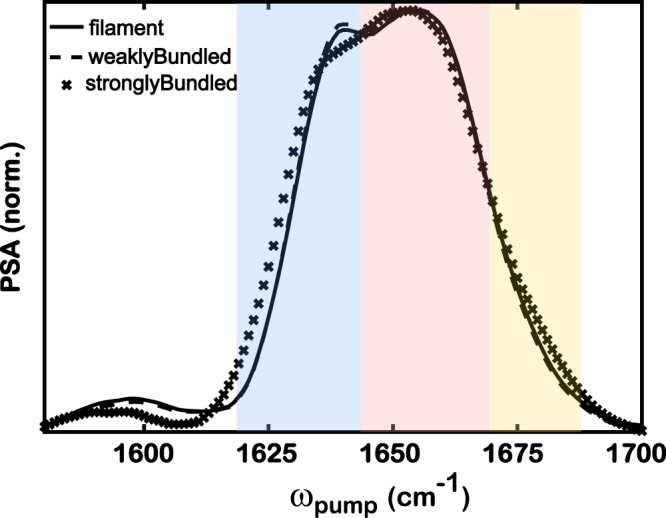
Pump slice amplitudes (PSA) calculated from 2D IR spectra capture the main features of secondary structures in three frequency regions. The spectra are normalized to the maximum intensity for the comparison among three samples. The pump frequency is plotted along the horizontal axis. Shaded areas indicate three spectral features, where blue, pink, and yellow represent approximate frequency regions for *β*-sheet, *α*-helix, and loop structure, respectively.

The 2D IR spectra offer insights into the backbone structure and conformational changes in F-actin across three different morphologies. One-dimensional FT IR spectra of these samples are particularly difficult to measure because of the low absorbance and strong background resulting from the dilute concentrations used. As an alternative, we perform a pump slice amplitude (PSA) analysis using 2D IR spectra to obtain features that are comparable to FT IR^77^. In short, PSA analysis projects a 1D spectrum from the 2D spectrum by computing the difference between the maxima and minima of a series of pump-probe slices that are cut through the 2D IR spectrum at each pump frequency. Here, 2D IR spectra at a delay time of 0.15 ps are chosen because, as described above, energy transfer does not significantly contribute to the measured signals at this early delay time. The PSA spectra are used for the direct comparison of amide I bands in different states of actin (Fig. 4). The amide I spectra can indicate different secondary structures, where *α*-helix gives rise to a band around 1650 cm^−1^, with *β*-sheet on the lower frequency side and a loop structure on the higher frequency side^71,73,74^. Similar peak features can be observed in the spectra of the actin networks (Fig. 4), where the strongest band at 1660 cm^−1^ indicates the major structure of *α*-helix. There is a side band at 1640 cm^−1^, indicating the presence of *β*-sheet structure in the actin networks. Furthermore, a minor feature likely to arise from the loop structure is observed, especially for the strongly bundled network sample, as indicated by the rising shoulder around 1680 cm^−1^. It is worth noting that the weak peak at 1590 cm^−1^ corresponds to the asymmetric carboxylate stretches of glutamate and aspartate residues^78,79^. The analysis of these bands is outside the scope of this study. Further interpretation of the amide I band of the secondary structures of actin networks can provide valuable information about the conformational differences between bundles and filaments.

### Depletion forces alter the backbone structure of F-actin

**Fig. 5:**
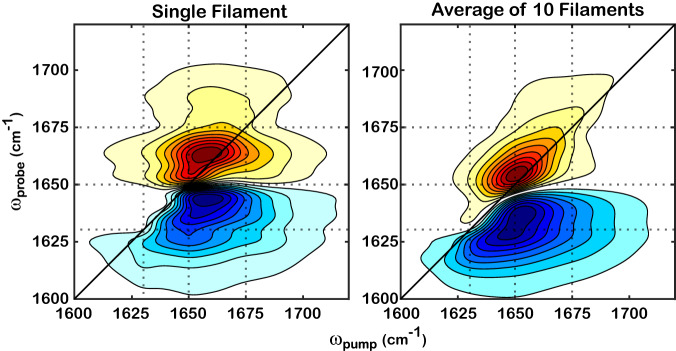
The computed 2D IR spectra capture the same features as those in the experiments. Amide I excitonic calculations are performed for a single-filament structure (left, PDB:*6BNO*) and 10 structures with site disorder (right, average of 10 renditions). As described in the text, the axes are shifted by 18 cm^−1^ to achieve an optimal match to the experimental spectrum. The solid black lines represent the diagonals of the 2D spectra. The dotted lines indicate the guidance of the frequency positions: 1630, 1650, and 1675 cm^−1^.

Spectral calculations are based on filament structures determined by cryo-electron microscopy (PDB file: *6BNO*) to further interpret the IR features of actin and validate the spectral assignment. The excitonic model reproduces the three main experimentally observed modes very well, allowing us to perform further analysis to explain the origin of the modes. However, it does not capture the elongation along the diagonal (Fig. 5), which results from the static inhomogeneity of the ensemble. To correct for this, we average 10 iterations with random local-mode frequencies drawn from a normal distribution, which results in a close match to the experimental spectra (Fig. 3). The diagonal traces of the computed 2D IR spectra are extracted to capture the main features of the lineshape (shaded areas in Supplementary Fig. 2), resulting in three similar main modes that are observed in the experimental spectra as shaded in Fig. 4. Note that PSA analysis is also performed for the calculated 2D IR, which does not resolve the three main modes clearly, likely because the anharmonicity between the two transitions and the computed inhomogeneous broadening in 2D IR are not strictly comparable to the experiments.

**Fig. 6:**
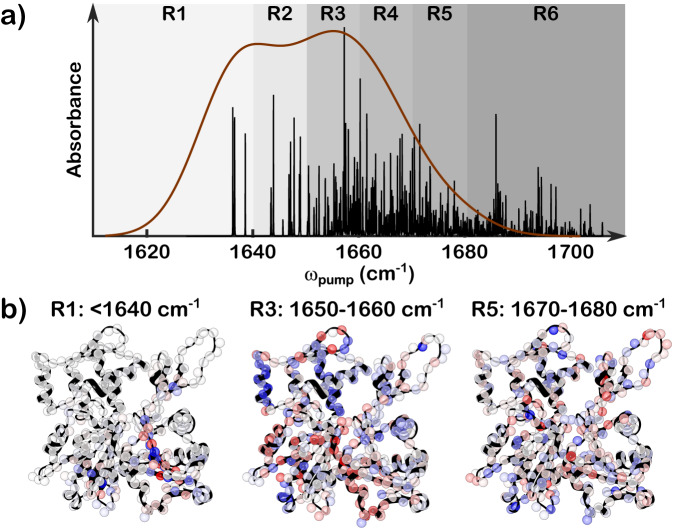
Doorway state analysis of amide I modes resolves the frequency-structure relation of the F-actin. **a** Experimental lineshape (brown solid line) overlaid with calculated IR modes (black sticks) of amide I stretch. The stick spectrum in black is generated by taking the square of the dot product of the eigenvectors of the one-exciton amide I Hamiltonian and the local amide I mode transition dipole moments, convoluted with Lorentzian lineshapes with a width of 0.01 cm^−1^. The shaded areas indicate six frequency regions for doorway state analysis, as listed in Table 1. **b** Visualization of monomer secondary structures and doorway state calculations for regions R1, R3, and R5 as indicated in the panel above. Amino acid residues are color-mapped to encode the amplitude and phase of each local amide I site within a given delocalized doorway state. Red and blue represent the positive and negative phases, respectively, where the intensity of the color encodes the amplitude of the amide I stretch. Sites that contribute little or none to a given state appear in white.

In Fig. 6, the stick spectrum that indicates the normal-mode intensities is overlaid with the experimental filament PSA lineshape, showing a qualitative match between the experiment and the computation. We apply a doorway state analysis^80,81^ to the calculated eigenmodes to visualize the backbone structures that contribute to different frequency ranges in the amide I region. As described in the “Methods” section, we divide the region into six frequency ranges, following the work of Chung and Tokmakoff^82^. The ranges R1–R6 are shaded in gradient colors (light to dark gray) in Fig. 6a. The contributions from three types of secondary structures, as defined in the *6BNO* PDB, are calculated for each frequency region and listed in Table 1. It is observed that R1 contains contributions almost exclusively from *β*-sheet when the R5 to R6 range has the most features of loop structures. The contribution from *α*-helix is spread out over the entire range but is the most dominant in R3. The amide I doorway modes are visualized on the actin structures in Fig. 6b), where the red to blue colors represent the positive to negative phases of vibrations in each frequency range, and the color depth encodes the vibration amplitude^82^. The structure-frequency correlation in the amide I spectrum is resolved, validating our peak assignment in 2D IR spectra. Following this assignment, the discrepancy in the spectra between the filament and the strongly bundled network (Fig. 4) demonstrates a conformational change upon morphological changes. The growth of the shoulder peak around 1680 cm^−1^ and the decrease in the *β*-sheet feature in the strongly bundled network indicate a fraction of *β*-sheets transitioning into loops, causing more disordered actin backbone structures. This finding seems to agree with the reported morphological changes driven by the depletion of PEG, where the bundle structures are found to be more heterogeneous than the pure filaments^65^.

**Table 1:** The secondary structure contributions to the amide I normal modes are resolved in the six frequency ranges derived from the doorway state analysis.

Region	Doorway range (cm^−1^)	Helix (%)	Sheet (%)	Loop (%)
R1	<1640	3.8	73.9	22.2
R2	1640–1650	36.4	20.2	43.4
R3	1650–1660	62.4	15.0	22.6
R4	1660–1670	56.7	12.3	31.1
R5	1670–1680	37.7	14.9	47.4
R6	>1680	25.0	13.4	61.6

Secondary structure assignments are obtained from the PDB file 6BNO. For simplicity, all non-helical and non-sheet residues are defined as loop structures.

**Fig. 7:**
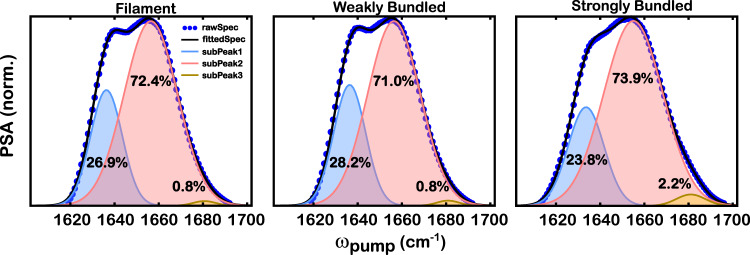
The fitting of the pump slice amplitude (PSA) with the three Gaussian profiles shows a decrease in *β*-sheet feature and an increase in the loop feature. The total area of each PSA spectrum is normalized to 1. The percentage of each sub-peak was calculated as the sub-peak area over the total area of each spectrum. Blue dots indicate the experimental PSA. The solid black line represents the fitted spectrum. Shaded areas indicate three-peak fittings, where blue, pink, and yellow represent *β*-sheet, *α*-helix, and loop structures, respectively. A bar plot of area percentages with error bars is provided in Supplementary Fig. 4. Notice that the error bars for area percentages in the loop region are within 0.5%.

The difference in the PSA spectral features provides evidence for conformational changes in the three backbone structures upon bundle formation. The change in the amide I band lineshape is quantified by fitting the PSA spectra to Gaussian profiles with three sub-peaks. The raw spectra, fitted spectra, and three fitted subpeaks are shown in Fig. 7. The fitting parameters are shown in Supplementary Fig. 3. Note that all fittings have the peak center of *α*-helix fixed, such that the parameters of *α*-helix are roughly kept the same across all samples. It is worth noting that there are likely to be more than three species of amide I stretch in this spectral region because actin backbone structures can form different numbers of hydrogen bonds with water. Here, we fit the spectra with three Gaussian profiles to avoid overfitting, so that each peak corresponds to one backbone structure, irrespective of the hydrogen-bonding species. The simplified model of the three Gaussian profiles (Fig. 7) can capture the overall PSA lineshape very well. The PSA interpretation is similar to that of FT IR spectra according to the Beer–Lambert law, but the transition dipole moments of the different amide I modes may be different; thus, it is not feasible to quantify secondary structure fractions based on IR lineshapes alone. Nonetheless, the differences in peak amplitudes and widths among the samples can be used to qualitatively describe the changes in the secondary structure across different morphologies, as well as the heterogeneity in the local environments. The full width at half maximum (FWHM) of the Gaussian peak for *α*-helix, *β*-sheet, and loop structures are 30, 18, and 13 cm^−1^, respectively. The broader width of *α*-helix can be assigned to a wider distribution of hydrogen-bond populations, where a larger width indicates multiple populations. The narrower width of *β*-sheet and loops indicates that they are more inclined to have a single species, for example, the one-hydrogen-bonded configuration, compared to the *α*-helix. The difference in FWHM between *α*-helix and *β*-sheet/loop is ~15 cm^−1^, which matches well with the observed redshifts of amide I stretch due to hydrogen bonds^83,84^. Another possible explanation for the broader linewidth is the slowdown of the dynamics in the local environment of *α*-helix, as discussed below.

The total area of each PSA spectrum is normalized to 1. The relative percentage of each subpeak is calculated as the subpeak area over the total area so that we can compare the differences among the three samples in Fig. 7. The area fraction of *α*-helix was ~72% with a 1% difference for all three samples, indicating the dominance of *α*-helical structure. The difference in the area percentage of *β*-sheet is statistically indistinguishable for filaments (27%) and weakly bundled networks (28%, Supplementary Fig. 4) but decreases slightly to 24% by 4% when strongly bundled networks are formed. Interestingly, the area percentage of the loop and turn structures is negligible and remains at 0.8% for filaments and weakly bundled networks, but it becomes more pronounced and almost triples (2%) when bundles become strong. The opposite trend of changes in the peak area between *β*-sheet and loop seems to indicate a correlation between the two backbone structures upon morphology change, where a fraction of *β*-sheets transitions into loops when the bundles are strong. However, the lineshape difference is relatively small for a concrete conclusion on the structural transition, considering that both the *β*-sheet and the loop are overpowered by the *α*-helix. Furthermore, the fitting of each structure with one Gaussian profile is a simplified model because there could be more than one hydrogen-bonded species under each fitted peak that would not be well fitted by a single Gaussian profile. Thus, the area percentage calculated from Gaussian fitting cannot be treated as a quantification of the change in the backbone population. Rather, it is a good indicator for the structural comparison of filamentous and bundled actin networks, providing evidence for the structural transition in the actin backbones. Further analysis of time-dependent 2D IR spectra in the following section will provide stronger evidence for the structural transition in the strongly bundled network. Overall, PSA analysis offers insights into the actin backbone structure undergoing morphological changes from filaments to strongly bundled networks, which implies a structural transition from *β*-sheet to loop structures.

### Dynamics analysis indicates less hydrated backbone in strongly bundled actin network

Fluctuations in the local environment of the actin backbone are primarily driven by hydrogen bonding, either to other residues in the proteins or to water molecules. Because water molecules undergo fast dynamics on a sub-picosecond timescale^85,86^, backbone fluctuations are faster in solvent-exposed regions than in residues that are locked in more rigid secondary structures, such as the protein core. Using waiting-time-dependent 2D IR spectra, we construct an evaluation of the dynamics timescale by analyzing the changes in lineshapes as a function of the waiting time or pump-probe delay (*t*_2_). As described in the “Methods” section, we use the center line slope method to represent changes in the 2D lineshapes^87^ and extract the dynamics of local environments for three secondary structures in different frequency regions: *α*-helix, *β*-sheet, and loops (see also Supplementary Figs. 5–7).

**Fig. 8:**
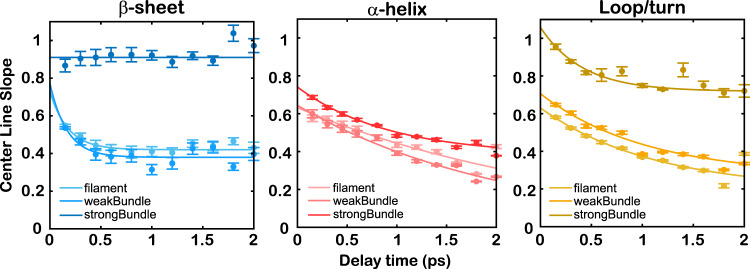
Analysis of the vibrational dynamics for each secondary structure indicates less hydration around the actin backbone in the strongly bundled network. The center line slope (CLS) is plotted versus the delay time (*t*_2_) between the pump and probe pulses. The error bars for CLS are the standard errors computed from the fittings of center lines using the linear regression model at each delay time. The solid lines represent exponential fitting curves, as described in the text. Light-to-dark colors represent filament to strongly bundled network samples. Details of fittings are provided in the “Methods” section. Center lines at different delay times are shown in Supplementary Figs. 5–7.

The results of the dynamics analysis are shown in Fig. 8. Note that data points at later time delays are more off-trend because of the lower IR signal resulting from the population relaxation. All the fitting parameters are listed in Supplementary Tables 1–3. The amplitude *A*_1_ is related to the amide I frequency fluctuations, where a larger amplitude indicates increased disorder in the local environment. The time constant *τ*_1_ represents the timescale of the motions within the local environment. The offset *y*_0_, also known as static inhomogeneity, is related to the solvent-inaccessible amide I modes, whose dynamics are slower than the investigated time window. It is worth noting that the offset from the fitting also depends on the selected frequency region because of the peak overlap of different backbone structures. Because we select three frequency regions to reveal the solvent dynamics around the three secondary structures, the offset from the fitting cannot be directly compared across the three structures. It is also discovered that the CLS of *β*-sheet decays very fast within 0.3 ps and plateaus afterward, making it difficult to accurately extract decay constants for *β*-sheet. Therefore, only the values of the offset *y*_0_ are listed for the *β*-sheet in Supplementary Table 2.

Overall, the 2D IR dynamics showed changes in the local environment across all the secondary structures in the strongly bundled networks. The dynamics are relatively static in the picosecond time regime, as shown by the presence of an offset of up to 2 ps (Fig. 8), which indicates a small solvent exposure along the actin backbone. The fitting results are similar for filamentous and weakly bundled actin networks, but different for strongly bundled networks, irrespective of the backbone secondary structure. This finding is consistent with the changes in the 2D IR lineshapes, where a different lineshape is observed for the strongly bundled network. Under the depletion effects of PEG, actin undergoes morphological changes to form bundles, which affect the degree of solvent exposure around the amide I mode along the backbone when the space between neighboring filaments is depleted, causing different dynamics in strongly bundled networks. The changes in dynamics for a strongly bundled network could also be affected, albeit less substantially, by the coupling degree among the amide I modes along the actin backbone (supported by the cross peaks in Fig. 3 and Supplementary Fig. 1). However, given the high similarities among the secondary structures of different morphologies, the coupling degree is probably kept constant, and thus an insubstantial factor for the changes in the dynamics of the strongly bundled network. Meanwhile, the energy transfer happens on the order of a few ps, evidenced by the cross peak growth within 2 ps (see Supplementary Fig. 1). If energy redistribution would be the main reason for CLS difference among three samples, a dynamical process within 2 ps should contribute to the CLS decay, which is not observed here (Fig. 8). Therefore, energy transfer is not a major factor for the CLS difference upon morphological changes.

From the analysis of different frequency regions, the effects of morphology change due to PEG depletion interactions can be investigated for three types of backbone structures: *α*-helix, *β*-sheet, and loop structures. Because of the presence of multiple bands in the amide I stretch region, the dynamics extracted from the 2D IR lineshape are used for a qualitative comparison among different actin morphologies rather than a quantitative analysis of water dynamics in the local environment. For *α*-helix and loop structures, there is a fast decay of 1–2 ps (Supplementary Tables 1 and 3), corresponding to the fluctuations in hydrogen-bonded water molecules around the actin amide I mode. These timescales compare favorably with measured water dynamics^76,88^. The primary structure, *α*-helix, displays a fast decay in 2 ps with a close-to-zero offset for the filament and weakly bundled network (Fig. 8, middle), indicating rapid hydrogen-bond exchange dynamics. In the strong-bundle sample, hydrogen bond fluctuation occurs in 1 ps, after which the environment is more restricted and undergoes other slower dynamical processes, as indicated by the presence of an offset. The dynamics analysis for *α*-helix demonstrates a more static environment in a strongly bundled network, likely due to the attractions among filaments and less solvent exposure along the backbone.

The dynamics can also be analyzed for the *β*-sheet features. In filaments and weakly bundled networks, there is an initial decrease in CLS within 0.5 ps and then a plateau at 0.4 (Fig. 8, left). Upon strongly bundled network formation, the lineshape does not exhibit changes throughout the entire measurement (Fig. 8 and Supplementary Table 2), indicating that the *β*-sheet structure is “locked” with very little solvent exposure in the surroundings. Such finding demonstrates that the *β*-sheet structure is mostly shielded from the solvent, especially in strongly bundled networks, and therefore very likely to distribute in the “buried” sites of actin networks. The interesting change in the dynamics of *β*-sheet provides great insight into its role in the mechanical and biochemical functions of actin bundles.

Finally, the loop structures share some similarities with *α*-helix and exhibit important differences across morphologies. In filaments and weakly bundled networks, the loops have features similar to those of the *α*-helix, with 1 ps dynamics and 0.2 offsets. In contrast, the CLS curve in strongly bundled networks has a fast decay within 0.3 ps and an offset afterward (Fig. 8, right), similar to the results of the *β*-sheet. Thus, loop structures are mostly exposed to free solvents in the filament and weakly bundled network but are almost constrained in “buried” positions with little solvent exposure in the strongly bundled network. The diverse features of loops from filaments to strongly bundled networks seem to indicate the important role of the disordered backbone in the physical and biochemical properties of actin networks.

### Crowded environments alter physical and biochemical properties of F-actin

We investigate the backbone structure and structure-dependent solvent exposure using ultrafast laser spectroscopy and interpret the lineshape changes from filaments to bundles using structure-based vibrational models. 2D IR spectroscopy also enables differentiating the roles of three secondary structures in actin networks under morphological control and helps form hypotheses of different contributions from *α*-helix, *β*-sheet, and loops. The secondary structure changes derived from the amide I lineshape (Fig. 7) and the dynamics measured by 2D IR spectra (Fig. 8) can be used to understand the role of the actin backbone in the physical and biochemical functions of the actin networks. In general, filaments and weakly bundled networks (bundling degree lower than 0.2^65^) share similar backbone structures and solvent environments, as evidenced by the similar amide I lineshape and dynamics across the two morphologies. The lineshape and dynamics for strongly bundled networks are different, suggesting changes in the local environment within this morphology.

First, excitonic calculations and doorway state analysis (Figs. 5 and 6) help the peak assignment in three frequency regions, each of which contains the major contribution from one type of secondary structure. Second, lineshape modeling shows more pronounced loop features accompanied by a decrease in the fraction of *β*-sheet in strongly bundled networks, indicating the structural transformation from *β*-sheet to loop/turn, although by a relatively small fraction. Such change in backbone structure could, in principle, arise from the space between filaments due to polymer-crowding. However, the inter-filament distance is the same for weakly and strongly bundled networks^65^. Thus, inter-strand interactions are unlikely to be the cause of changes in the loop region within the strongly bundled networks. Third, the increased features of the loops should demonstrate a more disordered or heterogeneous network in comparison to the filamentous sample, in agreement with dynamic-light-scattering measurements^65^. Lastly, the strongly bundled network exhibits lower solvent exposure due to the interactions between neighboring filaments, leading to more constrained solvent environments and slower hydrogen bond exchange rates. It is important to note that the changes observed in strongly bundled networks are likely to take place in cytoplasmic regions characterized by polymer-crowding. However, there are notable differences between our sample and actual cells. Cells undergo active molecular processes that result in a high turnover of structure and stress in the actin networks. Additionally, the effects of cross-link proteins on actin bundles, apart from polymer-crowding, have not been considered in the current framework. While similar changes in actin networks may occur in cytoplasmic environments, it is challenging to envision an identical actin structure within cells as observed in our study.

The sensitivity of the amide I modes helps gain access to the degree of hydration within the different backbone structures. Among the three secondary structures, *α*-helix is the dominant species, followed by the *β*-sheet and loops. As discussed above, the relative populations of different structures remain invariant across filaments and weakly bundled networks. However, for strongly bundled networks, there is a transition from *β*-sheet to loops corresponding to more disordered backbones, which can be associated with an increase in structural heterogeneity within the actin network. Compared to *β*-sheet and loop structures, *α*-helix features are the most preserved across morphologies (Figs. 7 and 8). As shown in the dynamics analysis, the *α*-helices of actin biopolymers are the most solvent-exposed, with fast water fluctuations in the local environment. In contrast, the *β*-sheet structure was shown to be another abundant backbone structure in the actin network, whose measured dynamics indicate a very static local environment (Fig. 8, left). Upon strongly bundled network formation, *β*-sheets in the actin backbones become increasingly rigid, as there are essentially no dynamics within the picosecond scale of the measurement. Because the dynamics measured by 2D IR report on the hydrogen bonding dynamics of local water molecules, static dynamics are explained as low exposure to solvents around *β*-sheets. This finding allows us to hypothesize that *β*-sheets are generally less solvent-exposed across all morphologies; in strongly bundled networks, they are shielded from water solvation by being buried in the bundle core. Based on this evidence, we also hypothesize that *β*-sheets of actin bundles play a minor role in protein binding or solvent exchange. The more static dynamics around *β*-sheets in the strongly bundled network can also result from more immobilized water molecules in the local environment. It is possible that the water molecules are more ordered and less mobile between *β*-sheets, leading to more static dynamics in 2D IR analysis^89^.

These findings suggest an essential role of loops in the physical and biochemical properties of bundled actin networks. Loops constitute only a small fraction of the residues, suggesting that actin is highly structured. The depletion interaction of PEG 20 kDa induces attraction among filaments, rearranging the backbones of F-actin, and causing part of the *β*-sheet to transition into loops in a strongly bundled network. In filaments and weakly bundled networks, the loop structure is exposed to solvent molecules, resulting in relatively fast hydrogen bonding dynamics in the local environment. High solvent exposure may be correlated with a high propensity to bind other proteins in solution. In fact, many actin-binding proteins interact with actin loops in monomers and filaments, such as the DNase-I loop and actin-binding cleft^46,90^. In strongly bundled networks, part of the *β*-sheet regions likely contains short loops, which gives rise to a static environment of loops in this morphology compared to filament and weakly bundled networks (Fig. 8, right). As discussed above, increasingly immobilized water can also contribute to more rigid environments of loops in strongly bundled networks^89^. It is likely that these extended loops are distributed across buried regions, which are more constrained and less solvent-exposed than the loops at the solvent interface. To this end, the spatial distribution of different secondary structures in crowded environments is deduced, where the hydration of the actin backbone is altered by polymer depletion interactions. It is worth noting that solely based on our findings, the effects of loops on the mechanical and biochemical properties of actin networks remain elusive. Nevertheless, all results in this work point towards the non-negligible role of the loop structure, which has a substantial impact on the functions of actin bundles despite its minor fraction in the backbone structures.

## Methods

### Sample preparation

The actin protein was purchased from Cytoskeleton Inc. (rabbit skeletal muscle, >95% pure, product number AKL99). All protein stocks were centrifuged at 100,000×*g* for 5 min upon reconstitution in deuterated water (D_2_O). These protein stocks were stored between 0 and 4 °C and used within 7 days. The G-actin concentration was determined by measuring the solution absorbance at 290 nm against a background solution of 50 mM KCl, 20 mM imidazole (pH 7.4) with a Nanodrop One (ThermoScientific, Wilmington, DE, USA) using extinction coefficients of 26,600 M^−1^ cm^−1^. Note that the actin was reconstituted in deuterated water (D_2_O) instead of water (H_2_O) to eliminate the strong vibrational water band (OH) in the amide I stretch region.

Polyethylene glycol (PEG) 6 and 20 kDa were purchased from Millipore Sigma (Product numbers 8.18897.1000 and 8.07491.1000). Potassium chloride (KCl), imidazole, dithiothreitol (DTT), magnesium chloride (MgCl_2_), and Adenosine triphosphate (ATP) were purchased from Sigma-Aldrich. Trolox was purchased from Acros Organics. Actin 10/17 filament samples were prepared with a buffer concentration of 20 mM imidazole (pH 7.4), 50 mM potassium chloride (KCl), 2 mM magnesium chloride (MgCl_2_), 1 mM dithiothreitol (DTT), 0.1 mM ATP, and 1 mM Trolox. All buffer solutions were prepared with deuterated water (D_2_O) to eliminate the strong water (OH) band in the amide I stretch region. The sample of actin bundles has an addition of 0.1% w/v (or 1 mg/mL) PEG with a molecular weight of 6 or 20 kDa to form a low or high degree of bundles, respectively, in buffer solutions. Note the mass per milliliter is equal for bundled samples, but the molecular weight between PEG molecules is different. Confocal microscopy revealed that a strongly bundled network occurs when the bundling degree is >0.2 [a.u.]^65^. The degree of bundling was shown to be below 0.2 for 0.1% PEG 6 kDa, and above 0.2 for 0.1% PEG 20 kDa^65^. In this study, the PEG 6 and 20 kDa samples are referred to as weakly and strongly bundled networks, respectively. All samples were freshly prepared, measured within 48 h, and stored at 4 °C before the measurements. The concentration of actin in all samples was adjusted to 1.5 mg/mL (36 μM) to produce a sufficiently high signal for 2D IR experiments but remained sufficiently low to remain within the biologically relevant regime. The actin samples are also below the concentration regime where actin organizes into nematic liquid^91^.

### Two-dimensional infrared (2D IR) spectroscopy

#### 2D IR optical setup

The ultrafast 2D IR optical setup has been described in detail previously^78^. In brief, a 1 kHz Ti:sapphire regenerative amplifier (Astrella, Coherent Inc.) was used to pump an OPA/DFG unit (Light Conversion Inc.) to generate 100-fs mid-IR pulses centered at 5.8 μm. The delay between the two pump pulses was generated using a Ge-based pulse shaper (Quickshape, PhaseTech Inc.). The time delay between the pump pulses (*t*_1_) was numerically Fourier transformed to generate the pump-frequency axis (*ω*_pump_ cm^−1^). The pump and probe pulses were overlapped onto the sample in a pump-probe geometry, after which the probe pulse spectrum was measured using a spectrograph equipped with a 128-pixel MCT array (Teledyne), and the probe frequency (*ω*_probe_ cm^−1^) was measured directly in the frequency domain. The delay between the two pump pulses was scanned from 0 to 3000 fs in 20 fs steps. The delay time between the pump and probe pulses (*t*_2_) was measured from 150 to 2000 fs, with 11 times in total. Each spectrum was averaged over 5 million laser shots to obtain a cleaner background. In addition to phase cycling, all spectra were measured under perpendicular polarization conditions to minimize scattered light at the detector. An illustration of the pump-probe 2D IR pulse configuration and measurements is shown in Fig. 2b).

#### Center line slope analysis

Time-dependent 2D IR spectra can be analyzed to evaluate the dynamics of the local environments around the actin secondary structure. The analysis of changes in 2D lineshapes as a function of the waiting time or pump-probe delay (*t*_2_) can provide a measure of the correlation time in frequency fluctuations. Here, we use the center line slope method to represent changes in the 2D lineshapes following previous work by Kwak et al.^87^, where the center line slope (CLS) is the slope of the ridge across the maxima of the amide I band. This slope typically decays exponentially with *t*_2_ as the correlation between the excitation and detection frequencies is lost as a result of frequency fluctuation and energy redistribution among the normal modes. In general, the time constants extracted from the evolution of 2D lineshapes report on the dynamics or fluctuations of local environments within different secondary structures. Three frequency ranges were selected to characterize the dynamics of the different backbone structures based on PSA analysis and doorway state analysis in the amide I spectrum (Fig. 7 and Table 1). The selected frequency ranges roughly match the ranges of R1, R3, and R5 in the doorway state analysis, where each frequency range covers the contribution primarily from one type of backbone structure. The center lines of the 2D IR spectra in the three frequency ranges are shown in Supplementary Figs. 5–7. Each center line is fitted using a linear regression model with 95% confidence intervals for the coefficients. The slopes of center lines are extracted from the fitting parameters of linear regression. The errors of slopes are computed as the standard errors of the corresponding coefficient. CLS versus *t*_2_ is fitted with a single exponential decay of the form:

1 $${\rm {CLS}}({t}_{2})={A}_{1}* {\rm {{e}}}^{-\frac{{t}_{2}}{{\tau }_{1}}}+{y}_{0},$$

where *A*_1_ represents the frequency fluctuation amplitude, *τ*_1_ is the correlation time constant, and *y*_0_ is the offset, sometimes denoted as static inhomogeneity, which is attributed to the amplitude of the dynamics that occur on a timescale longer than the experimental window.

### Spectral simulations

#### Excitonic calculations

All infrared spectra have been calculated based on the excitonic amide I approach originally developed for linear IR spectra by Krimm^92^ and Torii^80^, which was later extended to 2D IR spectra by Hamm and Hochstrasser et al.^93,94^. The calculation is implemented using the framework detailed in ref. ^95^. In short, the excitonic amide I Hamiltonian is constructed by analyzing the local modes and the couplings between them, calculated from the atomic coordinates in the PDB file of the actin filament (*6BNO*). The delocalized vibrational eigenmodes are obtained by solving the time-independent Schrödinger equation. The IR response of amide I can be calculated from the eigenmodes. In the case of the spectrum depicted in Fig. 5 and Supplementary Fig. 2, we include an inhomogeneous broadening of 10 cm^−1^ using the *gasdev* function of Numerical Recipes^96^ and an average of over 10 renditions. In this study, the unperturbed amide I local mode frequency employed to obtain an optimal match with the experiments is blue-shifted by 18 cm^−1^. In prior studies involving calculations for fibrinogen^97^, we found a similar blueshift, the reason for which remains unclear. Potentially, the typical hydrogen length is longer in filamentous proteins, such as actin and fibrinogen, than in globular proteins, for which a redshifted central frequency leads to a better spectral match, as is the case in refs. ^98–100^). Such elongated hydrogen bonds could be caused by internal strain due to the filamentous structure formation. Alternatively, a low hydration level of the proteins could also explain the observed blueshift with respect to ‘normally’ hydrated protein, as was observed for the dried films formed by cellulose-binding module (CBM), which required a similar blueshift in the spectral calculations to match the experimental spectra^101^.

#### Doorway state calculations

Following Torii and Tasumi^81,102^, the calculated spectra can be structurally analyzed using so-called *doorway state calculations*. This analysis is based on singular value decomposition (SVD) of the ensemble of *N* normal modes within a particular frequency range, which decomposes this ensemble into *N* orthogonal left and right singular vectors. These SVD components are sorted by their singular values, which reflect the relative contributions of various orthogonally decomposed components to the ensemble of normal modes within a particular frequency range. The first component is used as the doorway state, which is a characteristic of the modes in this range. As for all doorway states, it contains the most common features within the range due to the singular-value sorting of the doorway states. We divide the amide I frequency range (1600–1700 cm^−1^) into the same six doorway ranges used by Chung and Tokmakoff^82^, where the range between 1640 and 1680 cm^−1^ is divided into 10 cm^−1^ intervals (labeled as R2, R3, R4, R5 in the order of increasing frequency) when the range below 1640 cm^−1^ (R1) and above 1680 cm^−1^ (R6) form two groups. The splitting of the frequency range is guided by the density of eigenstates and the desired frequency resolution so that the number of eigenstates in each region is appropriate for resolving the frequency–structure relationship. For actin, in these six ranges, the first doorway states contain 42–70% of the squared transition dipole moments of the total of three bright doorway states. We pictorially represent the common features of a given range (see Fig. 6b) with the first column (which represents the first doorway state) of the *transformation matrix* given by the dot product of the eigenvectors and the left singular vectors of the SVD. This matrix can also be used to determine the relative contributions of different types of secondary structures by multiplying the squares of the first column of the matrix by binary vectors that list the presence of secondary structures as a function of the sequence index^82^ (see Table 1).

### Statistics and reproducibility

2D data were replicated from two different batches of samples prepared under the same conditions. Supplementary Fig. 8 shows the replicated 2D data at a delay time of 0.15 ps for comparison. Results indicate that different batches of samples revealed the same lineshape changes from filaments or weakly bundled networks, to strongly bundled networks, confirming the reproducibility of spectral measurements.

### Reporting summary

Further information on research design is available in the Nature Portfolio Reporting Summary linked to this article.

### Supplementary information

Supplementary Information

Reporting Summary

## Data Availability

The structure of the actin filament can be found in the protein data bank with PDB ID: *6BNO*. All dataset analyses are available via the Texas Data Repository. (10.18738/T8/9ZDJW6, see reference^103^) All unprocessed datasets generated during the study are available from the corresponding author on reasonable request.

## Code availability

2D IR spectra were plotted and analyzed in MATLAB R2022B. Analysis codes for 2D IR spectra are stored in the Texas Data Repository^103^.
