# Supplementary material for: Crowding alters F-actin secondary structure and hydration
Source: Commun Biol. 2023 Sep 2;6:900. doi: 10.1038/s42003-023-05274-3 (PMC10475093; doi:10.1038/s42003-023-05274-3)
Supplement: Supplementary file 1 — Supplementary Information [file 42003_2023_5274_MOESM1_ESM.pdf]

Supplementary Information: Crowding alters F-actin secondary structure and hydration

Xiaobing Chen[1], Steven J. Roeters[2,3], Francis Cavanna[4], José Alvarado[4], and Carlos R. Baiz[1,*]

[1]University of Texas at Austin, Department of Chemistry, Texas, Austin, USA
[2]Aarhus University, Department of Chemistry, Aarhus, Denmark
[3]Vrije Universiteit, Department of Anatomy and Neurosciences, Amsterdam UMC, Amsterdam, Netherlands
[4]University of Texas at Austin, Department of Physics, Center for Nonlinear Dynamics, Texas, Austin, USA
[*]cbaiz@cm.utexas.edu

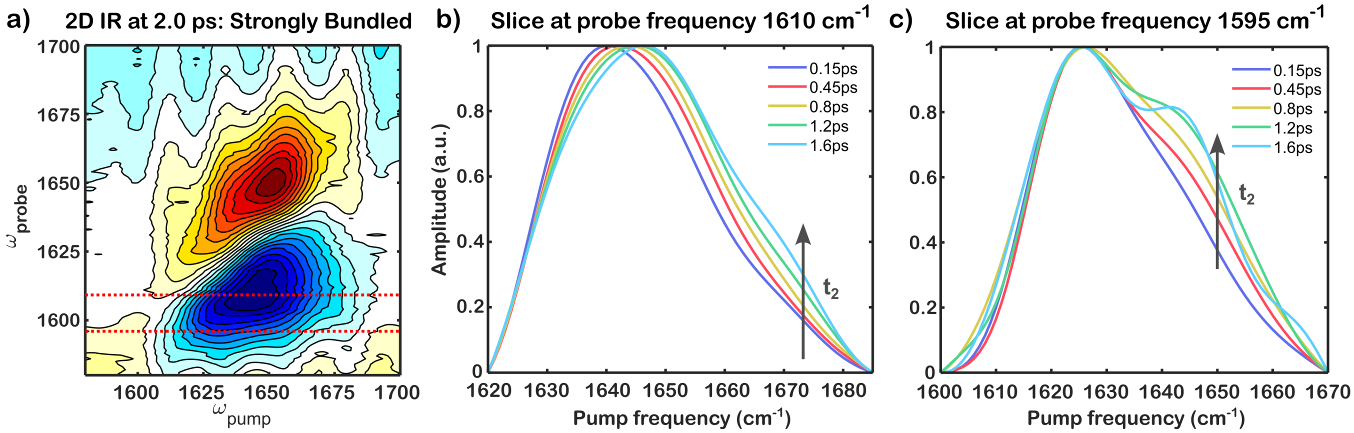

Supplementary Figure 1. Cross peak growth extracted from 2D IR for strongly bundled network. a) 2D IR of strongly bundled actin at 2.0 ps. Red dashed lines across pump axis represent the probe frequencies of 1610 and 1595 cm^{-1}. b) A slice cut at probe frequency 1610 cm^{-1} across pump frequency from 1620 to 1685 cm^{-1}. A shoulder at 1675 cm^{-1} grows with time. c) A slice cut at probe frequency 1595 cm^{-1} across pump frequency from 1600 to 1670 cm^{-1}. A shoulder at 1650 cm^{-1} grows with time.

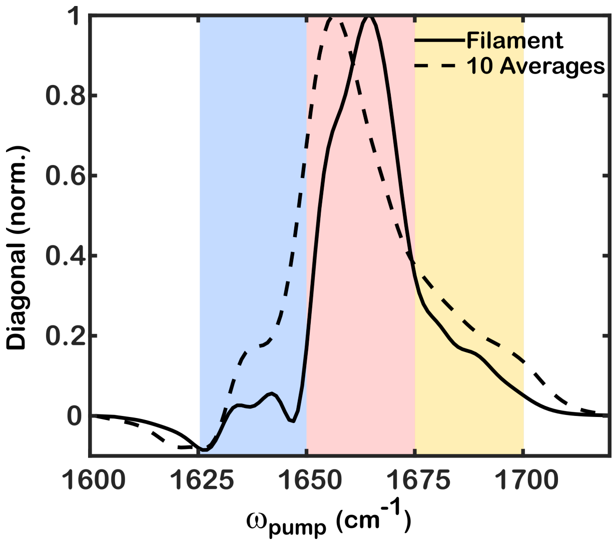

Supplementary Figure 2. Diagonal slices of calculated 2D IR spectra from excitonic calculation. Shaded areas represent the frequency ranges of different secondary structures. Blue, pink and yellow colors represent the regions containing contributions mainly from β-sheet, α-helix, and loop structures, respectively.

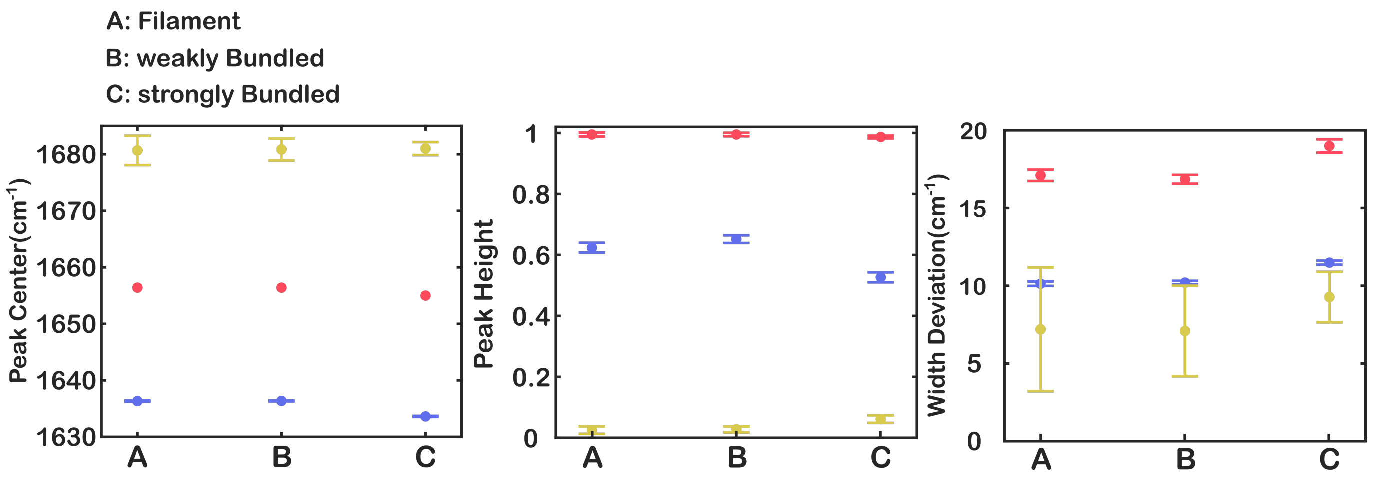

Supplementary Figure 3. Fitting parameters (peak center, peak height, and width deviation) from Gaussian fitting of Pump Slice Amplitude. Each PSA is fitted by three Gaussian sub-peaks. The error bars of fitting parameters are computed from the Gaussian fittings using non-linear least squares method with 95% confidence level. Blue, pink and yellow colors represent β-sheet, α-helix, and loop structures, respectively. In each plot, from left to right, there indicates the results of filamentous, weakly bundled, and strongly bundled networks.

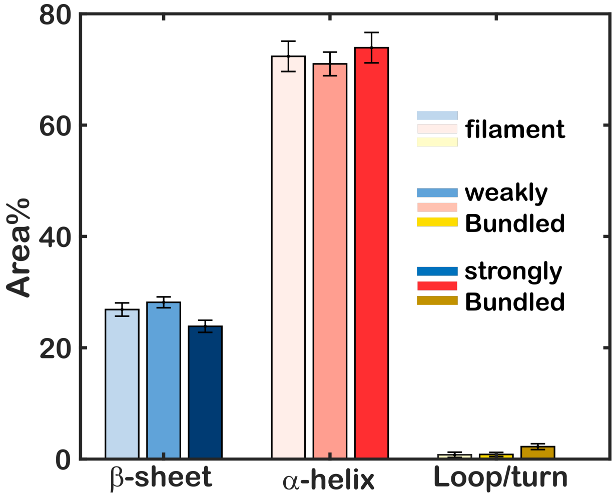

Supplementary Figure 4. Barplot of area percentages calculated from Gaussian fitting of Pump Slice Amplitude (PSA). Each PSA is calculated from the 2D IR spectrum at 150 fs, which was averaged over 5 million laser shots in 2 hrs. The peak area is the area of fitted Gaussian peak, which is computed using the Gaussian fitting parameters. The error bars for the areas are propagated from the Gaussian fitting parameters, as shown in Supplementary Figure 3. The total area of three backbone structures for each sample is 100%. The area percentage is defined as the sub-peak area over the total area of each spectrum. Blue, pink and yellow colors represent β-sheet, α-helix, and loop structures, respectively. Gradient colors are to indicate different morphologies. From light to dark colors, there indicate filamentous, weakly bundled and strongly bundled networks.

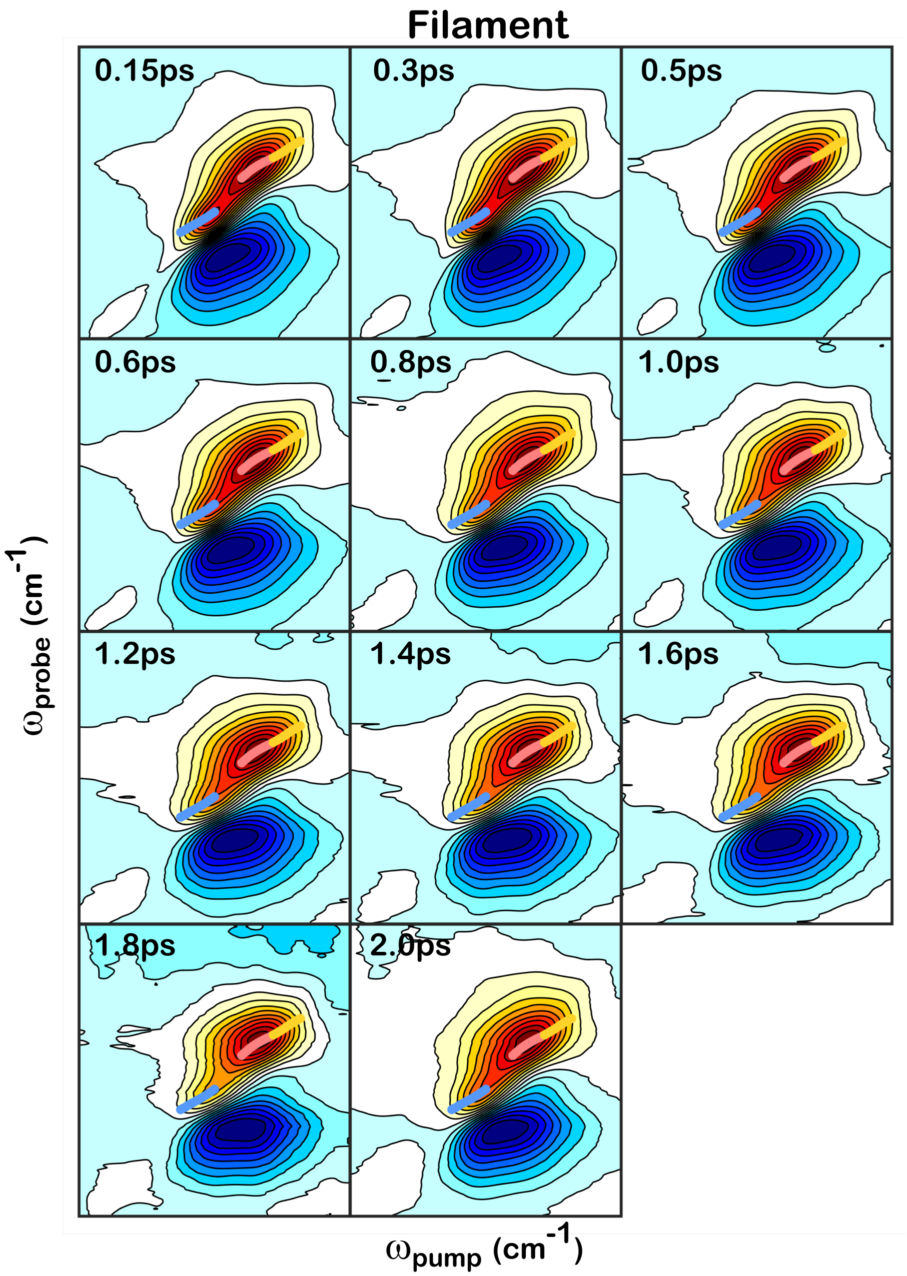

Supplementary Figure 5. Dynamics analysis of 2D IR spectra for filaments from 0.15 to 2 ps. Blue, pink, and yellow solid lines represent the analyzed frequency region for β-sheet, α-helix, and loop structures, respectively.

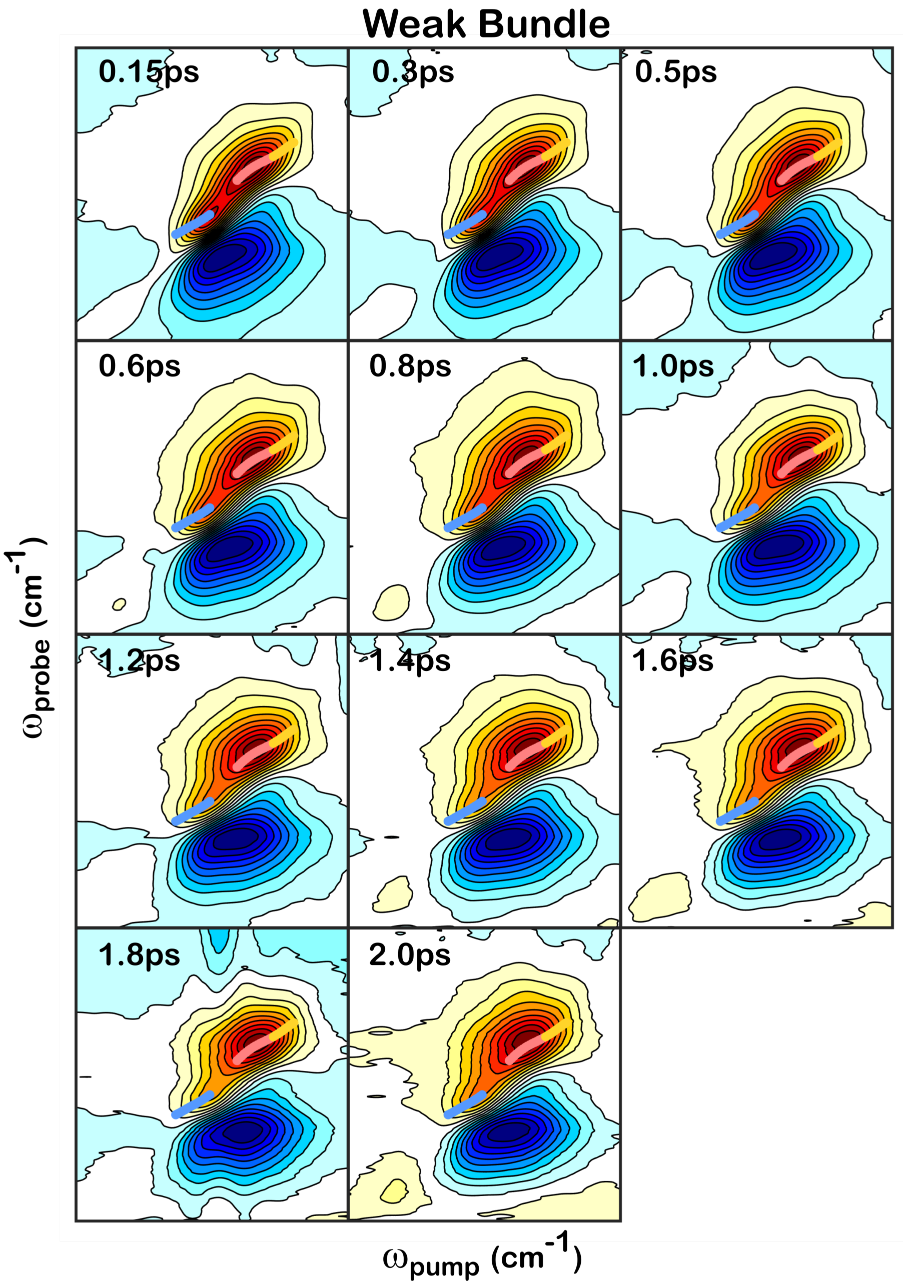

Supplementary Figure 6. Dynamics analysis of 2D IR spectra for weakly bundled network from 0.15 to 2 ps. Blue, pink, and yellow solid lines represent the analyzed frequency region for β-sheet, α-helix, and loop structures, respectively.

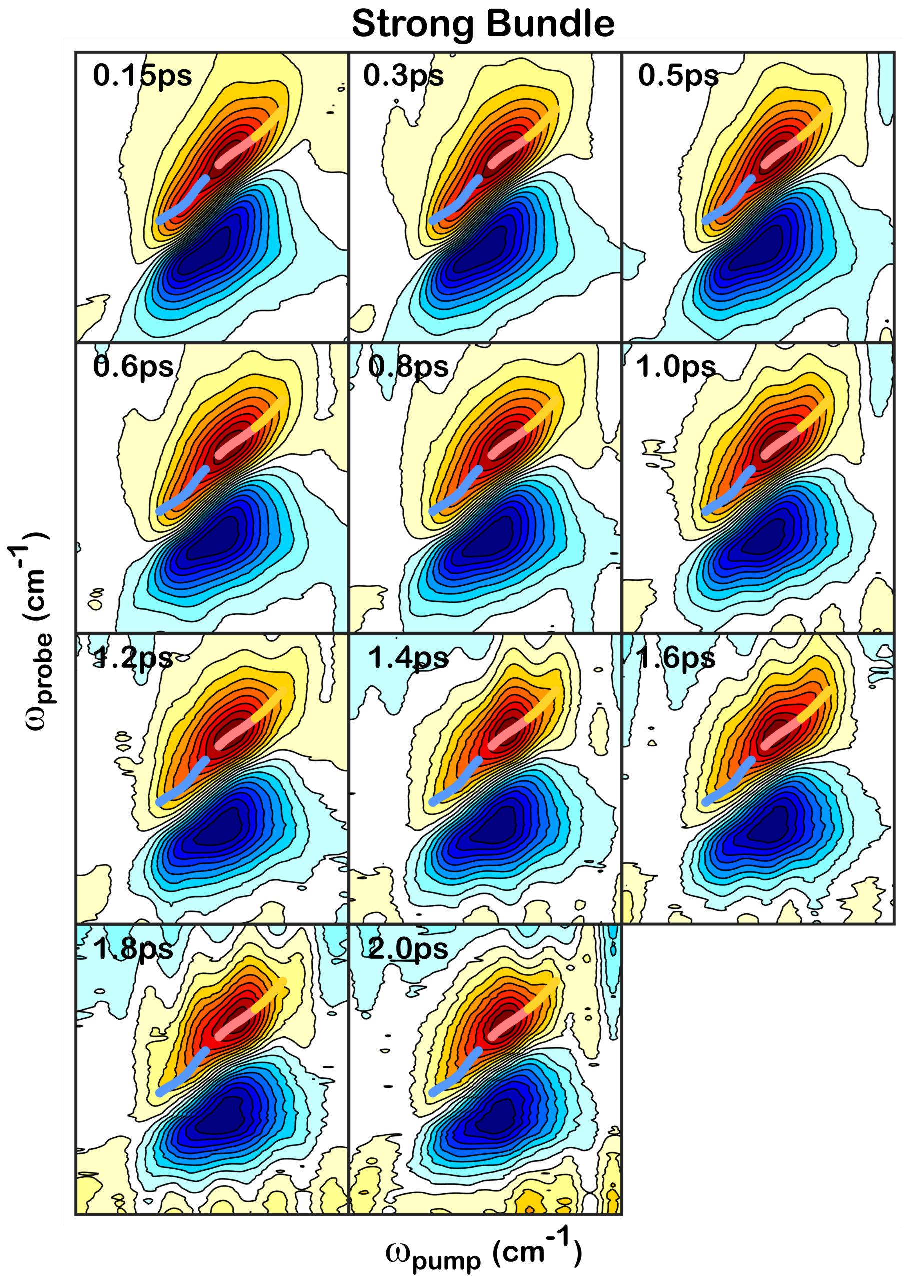

Supplementary Figure 7. Dynamics analysis of 2D IR spectra of strongly bundled network from 0.15 to 2 ps. Blue, pink, and yellow solid lines represent the analyzed frequency region for β-sheet, α-helix, and loop structures, respectively.

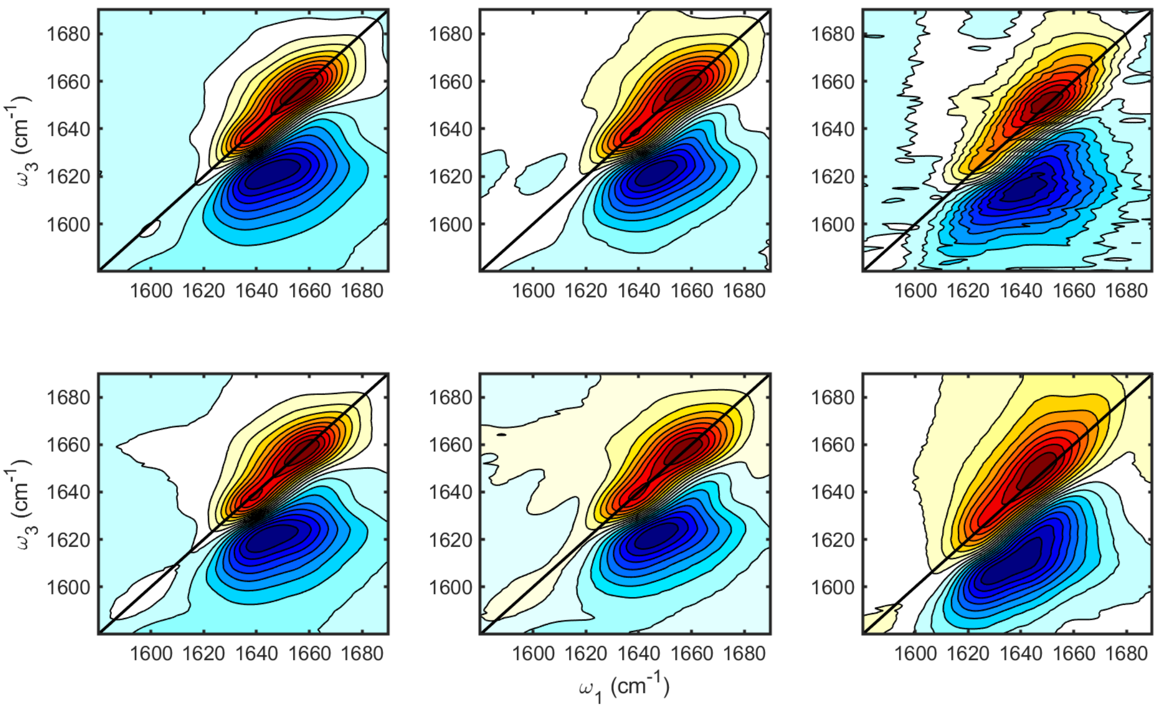

Supplementary Figure 8. 2D IR spectra of filaments (left), weakly bundled network (middle), and strongly bundled network (right) for two batches of samples prepared under the same conditions. Only the spectra at a delay time of 150 fs are listed here for comparison. Top and bottom panels show the datasets collected for first and second batches of samples, respectively.

Supplementary Table 1. Dynamics analysis for the center line slope of α-helix, fitted by a single exponential decay. The parameters (fluctuation amplitude, decay constant, and offset) are listed from left to right. The errors were computed from the single exponential fitting of the form:

$$CLS(t_2) = A_1 * e^{-\frac{t_2}{\tau_1}} + y_0.$$

We used the *fit* function with Robust linear least-squares fitting method in MATLAB.

α-helix	Filament	weaklyBundled	stronglyBundled
A_1	0.63±0.03	0.64±0.04	0.36±0.07
τ_1	2.85±0.49	2.10±0.33	0.91±0.59
y_0	0	0	0.38±0.10

Supplementary Table 2. Dynamics analysis for the center line slope of β-sheet, fitted by a single exponential decay. The parameters (fluctuation amplitude, decay constant, and offset) are listed from left to right. Errors were computed in the same way as Supplementary Table 1.

β-sheet	Filament	weaklyBundled	stronglyBundled
A_1		-	
τ_1		-	
y_0	0.42±0.03	0.38±0.05	0.92±0.05

Supplementary Table 3. Dynamics analysis for the center line slope of loops, fitted by a single exponential decay. The parameters (fluctuation amplitude, decay constant, and offset) are listed from left to right. Errors were computed in the same way as Supplementary Table 1.

loops	Filament	weaklyBundled	stronglyBundled
A_1	0.44±0.07	0.43±0.11	0.34±0.11
τ_1	1.12±0.43	1.03±0.91	0.42±0.26
y_0	0.19±0.08	0.27±0.15	0.72±0.04
