# Supplementary material for: Crowding alters F-actin secondary structure and hydration
Source: Commun Biol. 2023 Sep 2;6:900. doi: 10.1038/s42003-023-05274-3 (PMC10475093; doi:10.1038/s42003-023-05274-3)
Supplement: Supplementary file 2 — Reporting Summary [file 42003_2023_5274_MOESM2_ESM.pdf]

nature portfolio

Corresponding author(s): Carlos Baiz

Last updated by author(s): Aug 20, 2023

Reporting Summary

Nature Portfolio wishes to improve the reproducibility of the work that we publish. This form provides structure for consistency and transparency in reporting. For further information on Nature Portfolio policies, see our Editorial Policies and the Editorial Policy Checklist.

Statistics

For all statistical analyses, confirm that the following items are present in the figure legend, table legend, main text, or Methods section.

n/a	Confirmed	
☒	☐	The exact sample size (*n*) for each experimental group/condition, given as a discrete number and unit of measurement
☒	☐	A statement on whether measurements were taken from distinct samples or whether the same sample was measured repeatedly
☒	☐	The statistical test(s) used AND whether they are one- or two-sided<br>*Only common tests should be described solely by name; describe more complex techniques in the Methods section.*
☒	☐	A description of all covariates tested
☒	☐	A description of any assumptions or corrections, such as tests of normality and adjustment for multiple comparisons
☒	☐	A full description of the statistical parameters including central tendency (e.g. means) or other basic estimates (e.g. regression coefficient) AND variation (e.g. standard deviation) or associated estimates of uncertainty (e.g. confidence intervals)
☒	☐	For null hypothesis testing, the test statistic (e.g. *F*, *t*, *r*) with confidence intervals, effect sizes, degrees of freedom and *P* value noted<br>*Give P values as exact values whenever suitable.*
☒	☐	For Bayesian analysis, information on the choice of priors and Markov chain Monte Carlo settings
☒	☐	For hierarchical and complex designs, identification of the appropriate level for tests and full reporting of outcomes
☒	☐	Estimates of effect sizes (e.g. Cohen's *d*, Pearson's *r*), indicating how they were calculated

Our web collection on statistics for biologists contains articles on many of the points above.

Software and code

Policy information about availability of computer code

Data collection	Datasets collected in this study are deposited in Texas Data Repository as cited in reference 103.
Data analysis	Analysis codes for 2D IR spectra in this study are deposited in Texas Data Repository as cited in reference 103.

For manuscripts utilizing custom algorithms or software that are central to the research but not yet described in published literature, software must be made available to editors and reviewers. We strongly encourage code deposition in a community repository (e.g. GitHub). See the Nature Portfolio guidelines for submitting code & software for further information.

Data

Policy information about availability of data

All manuscripts must include a data availability statement. This statement should provide the following information, where applicable:

- Accession codes, unique identifiers, or web links for publicly available datasets
- A description of any restrictions on data availability
- For clinical datasets or third party data, please ensure that the statement adheres to our policy

The structure of actin filament can be found in protein data bank with PDB ID: 6BNO. The analyzed datasets generated during the current study are deposited in Texas Data Repository as cited in reference 103. Unanalyzed data are available from the corresponding author on reasonable request. All dataset analysis are shown in the paper and supplementary.

Human research participants

Policy information about studies involving human research participants and Sex and Gender in Research.

Reporting on sex and gender	No human research in current study.
Population characteristics	No human research in current study.
Recruitment	No human research in current study.
Ethics oversight	No human research in current study.

Note that full information on the approval of the study protocol must also be provided in the manuscript.

Field-specific reporting

Please select the one below that is the best fit for your research. If you are not sure, read the appropriate sections before making your selection.

☒ Life sciences ☐ Behavioural & social sciences ☐ Ecological, evolutionary & environmental sciences

For a reference copy of the document with all sections, see nature.com/documents/nr-reporting-summary-flat.pdf

Life sciences study design

All studies must disclose on these points even when the disclosure is negative.

Sample size	All protein samples were purchased and prepared under the same condition and following the same procedure as described in Methods section. Each spectrum was averaged over 5 million laser shots to ensure reproducibility and obtain a high signal-to-noise ratio.
Data exclusions	No data were excluded.
Replication	Each spectrum was averaged over 5 million laser shots to ensure consistency. Two batches of samples were measured for replicates of 2D IR spectra. All attempts to replication were successful.
Randomization	Randomization is irrelevant to this study. All protein samples were purchased and prepared under the same condition and following the same procedure. Two batches of samples were tested for replication. Experiments were repeated and averaged to ensure reproducibility and obtain a high signal-to-noise ratio.
Blinding	Blinding was irrelevant to this study. All protein samples were purchased and prepared under the same condition and following the same procedure. Experiments were repeated and averaged to ensure reproducibility and obtain a high signal-to-noise ratio.

Reporting for specific materials, systems and methods

We require information from authors about some types of materials, experimental systems and methods used in many studies. Here, indicate whether each material, system or method listed is relevant to your study. If you are not sure if a list item applies to your research, read the appropriate section before selecting a response.

Materials & experimental systems

n/a	Involved in the study	
☒	☐	Antibodies
☒	☐	Eukaryotic cell lines
☒	☐	Palaeontology and archaeology
☒	☐	Animals and other organisms
☒	☐	Clinical data
☒	☐	Dual use research of concern

Methods

n/a	Involved in the study	
☒	☐	ChIP-seq
☒	☐	Flow cytometry
☒	☐	MRI-based neuroimaging
